# Supplementary material for: Hypomyelinated vps16 Mutant Zebrafish Exhibit Systemic and Neurodevelopmental Pathologies
Source: Int J Mol Sci. 2024 Jul 1;25(13):7260. doi: 10.3390/ijms25137260 (PMC11242861; doi:10.3390/ijms25137260)
Supplement: Supplementary file 1 [file ijms-25-07260-s001.zip › ijms-3003245-supplementary.pdf]

Hypomyelinated *vps16* mutant zebrafish exhibit systemic and neurodevelopmental pathologies

Shreya Banerjee[1], Shivani Bongu[1], Sydney P. Hughes[1], Emma K. Gaboury[1], Chelsea E. Carver[1], Xixia Luo[1], Denise A. Bessert[1], Ryan Thummel[1]*

[1]Department of Ophthalmology, Visual and Anatomical Sciences, Wayne State University School of Medicine, Detroit, MI 48201

***Correspondence:** rthummel@med.wayne.edu

Supplemental Figures

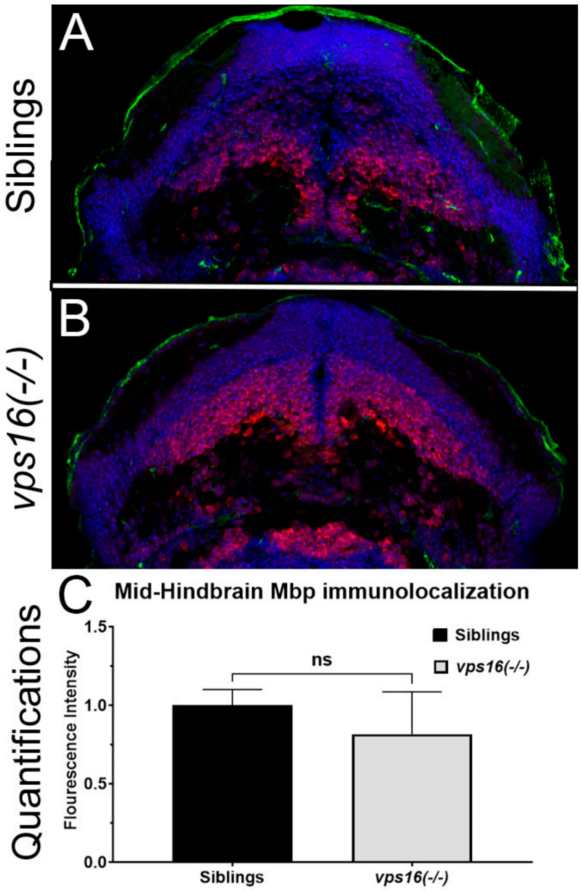

Supplemental Figure S1. *vps16(-/-)* mutants show no defect in myelination in the Mid-Hindbrain at 5 dpf. (A-B) Immunolocalization of MBP (myelin, green) and HuCD (neurons, red) in brain section from the Mid-Hindbrain region of siblings (**A**; n=3) and *vps16(-/-)* mutant larvae (**B**; n=3). **(C)** Graph representing quantification of MBP immunolocalization in the Mid-Hindbrain region at 5 dpf (p=0.5745). “ns” is not significant and error bars indicate SEM.

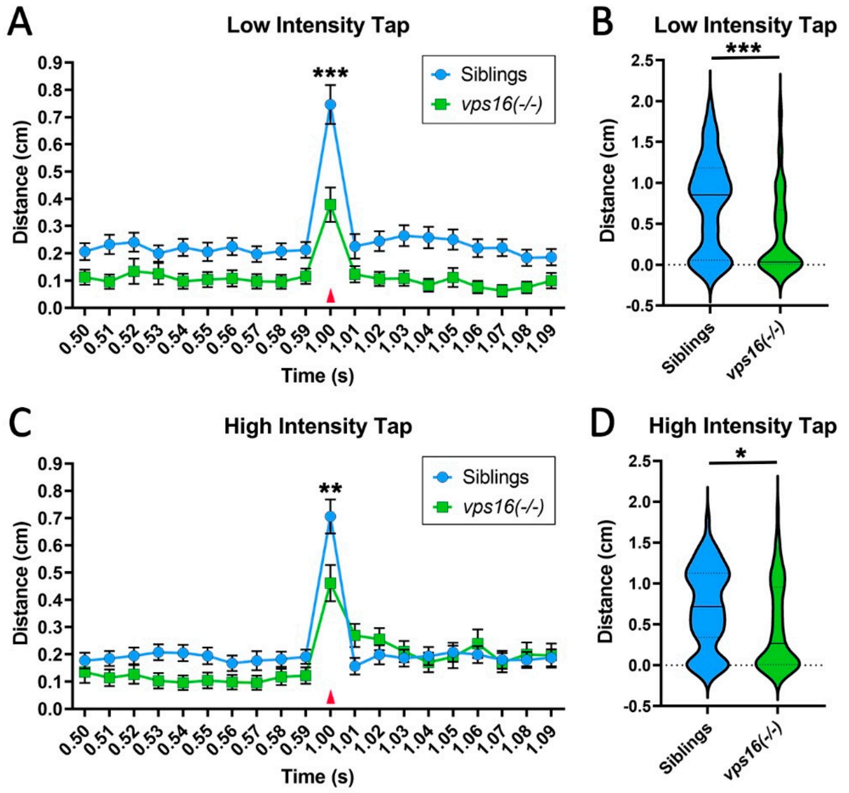

Supplemental Figure S2. *vps16(-/-)* mutants at 5 dpf show significantly reduced distance travelled in response to a single acoustic/tap stimulus at both low and high intensity. (A) Line graph representing average distance travelled by *vps16(-/-)* larvae (green, n=63) and siblings (blue, n=63) at 5 dpf in response to a single low intensity acoustic/tap stimulus (red arrow). P=0.0003. **(B)** Violin plots representing average distance travelled by *vps16(-/-)* larvae (green, n=63) and siblings (blue, n=63) at 5 dpf in response to a single low intensity acoustic/tap stimulus. **(C)** Line graph representing average distance travelled by *vps16(-/-)* larvae (green, n=62) and siblings (blue, n=63) at 5 dpf in response to a single high intensity acoustic/tap stimulus (red arrow). **(D)** Violin plots representing average distance travelled by *vps16(-/-)* larvae (green, n=62) and siblings (blue, n=63) at 5 dpf in response to a single high intensity acoustic/tap stimulus. For all graphs, “*” = P<0.05, “**” = P<0.01, “***” = P<0.001, and error bars indicate SEM.

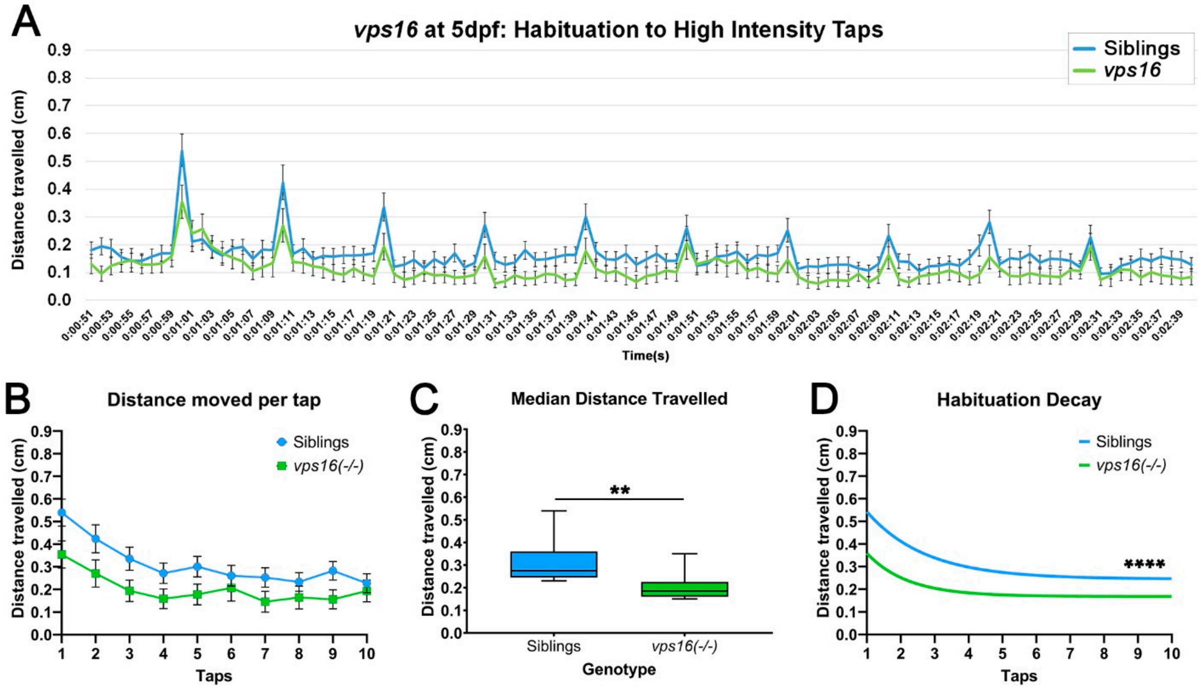

Supplemental Figure S3. *vps16(-/-)* mutants at 5 dpf show a significantly reduced habituation response to multiple high intensity acoustic/tap stimuli compared with their siblings. (A) Line graph representing distance travelled by *vps16 (-/-)* larvae at 5 dpf during the complete high intensity multiple tap paradigm comprising 10 high intensity taps with 10 s interstimulus intervals (ISI) (sibling in blue, *vps16(-/-)* in green). **(B)** Graph representing average distance moved by 7 dpf sibling (blue; n=59) and *vps16(-/-)* (green; n=55) larvae at each individual tap stimuli. **(C)** Box-plots representing the median distance travelled by 7 dpf sibling (blue; n=59) and *vps16(-/-)* (green; n=55) larvae in response to multiple tap stimuli. P<0.01 **(D)** First order exponential decay curve representing average distance travelled by 7 dpf sibling (blue; n=59) and *vps16(-/-)* (green; n=55) larvae at each individual tap stimuli. For all graphs, "**" = P<0.01, "****" = P<0.0001, and error bars indicate SEM.

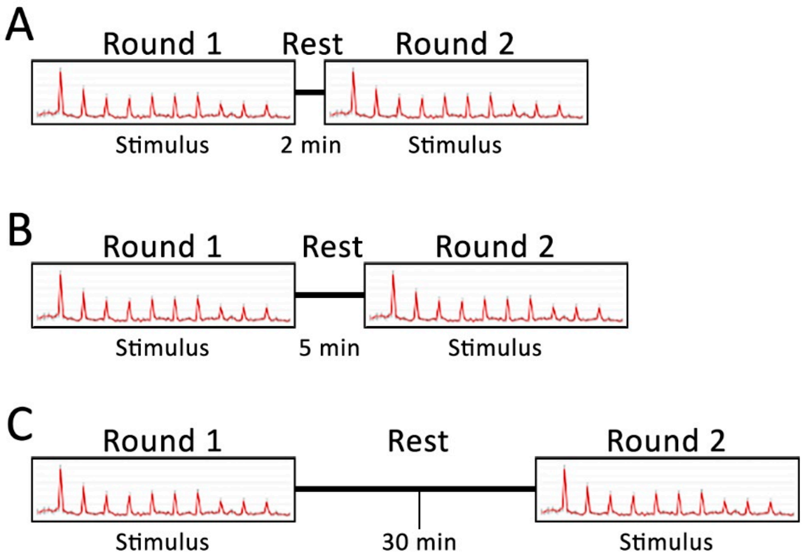

Supplemental Figure S4. Schematic representation of intermediate memory paradigms. (A) Schematic representation of intermediate memory paradigm showing round one stimulus having 10 high intensity taps, followed by a 2 min rest period with no stimulation, and finally round two stimulus having 10 high intensity taps. **(B)** Schematic representation of intermediate memory paradigm showing a 5 min rest period with no stimulation in between round one and round two stimulus sets having 10 high intensity taps each. **(C)** Schematic representation of intermediate memory paradigm showing a 30 min rest period with no stimulation in between round one and round two stimulus sets having 10 high intensity taps each.
